# Hybrid Coordination Networks for Removal of Pollutants from Wastewater

**DOI:** 10.3390/ijms232012611

**Published:** 2022-10-20

**Authors:** Marko Marganovici, Bianca Maranescu, Aurelia Visa, Lavinia Lupa, Iosif Hulka, Vlad Chiriac, Gheorghe Ilia

**Affiliations:** 1 Department of Biology-Chemistry, Faculty of Chemistry, Biology, Geography, West University Timisoara, 16 Pestalozzi Street, 300115 Timisoara, Romania; 2 “Coriolan Dragulescu” Institute of Chemistry, Romanian Academy, 24 Mihai Viteazul Bvd., 300223 Timisoara, Romania; 3 Faculty of Industrial Chemistry and Environmental Engineering, Politehnica University Timisoara, 6 Vasile Parvan Blv., 300223 Timisoara, Romania; 4 Research Institute for Renewable Energy, Politehnica University of Timişoara, G. Muzicescu 138, 300501 Timişoara, Romania

**Keywords:** metal phosphinates, adsorption, cadmium, lead

## Abstract

The adsorption properties of two coordination polymers, resulting from the reaction of divalent metal (Ca^2+^ or Co^2+^) salts with (2-carboxyethyl)(phenyl)phosphinic acid, are presented in this paper. The structural and textural characterization before and after adsorption experiments is presented. The adsorbent materials were prepared using the hydrothermal procedure. The compound Ca[O_2_P(CH_2_CH_2_COOH)(C_6_H_5_)]_2_ (CaCEPPA) has a layered topology, with the phenyl groups oriented into the interlayer space and crystallizes in the monoclinic system. Compound Co_2_[(O_2_P(CH_2_CH_2_COO)(C_6_H_5_)(H_2_O)]_2_·2H_2_O (CoCEPPA) has a 1D structure composed of zig-zag chains. The adsorption performances of CaCEPPA and CoCEPPA materials were tested in the removal of cadmium and lead from aqueous solutions. The optimum pH of ions adsorption was found to be five for both adsorbent materials. Pseudo-first and second-order kinetic models were used for fitting kinetic experimental data, and Langmuir and Freundlich isotherms were used for modeling the equilibrium experimental data. The pseudo-second-order kinetic model and Langmuir isotherm best described the adsorption of Cd and Pb ions onto the studied materials, judging from the results of the error function (correlation coefficient, sum of square error, chi-square test, and average relative error) analysis. The studied materials present a higher affinity for Cd ions compared with the adsorption capacity developed for the removal of Pb ions from aqueous solutions. CoCEPPA showed the highest adsorption performance in the removal process of metal ions from aqueous solutions compared with CaCEPPA (q_m_ = 54.9 mg Cd^2+^/g of CoCEPPA, q_m_ = 36.5 mg Cd^2+^/g of CaCEPPA).

## 1. Introduction

The danger posed to humans and the environment by extremely toxic heavy metal lead and cadmium ions in the environment remains a serious issue. Lead and cadmium ions are discharged into the environment from various industries such as leather, cosmetics, electronics, and battery manufacturing. Increased amounts of Pb(II) in the human body are very toxic because Pb(II) is non-biodegradable. It disturbs nearly all body systems and produces irremediable diseases, including heart disease, hematological problems, renal dysfunction, brain harm, cancer, and even death [1,2,3]. Cadmium ions Cd(II) can cause lesions in many major organs, including the kidney, and also cause bronchiolitis, pulmonary disease, fibrosis, and skeletal damage [4,5,6]. Various methods have been used for the elimination of Pb(II) and Cd(II), such as ion exchange, adsorption, coagulation-flocculation, chemical precipitation, electro-dialysis, and biological methods [7,8].

Among these, adsorption is the most used technique due to its low cost and easy working conditions [9,10].

Porous coordination polymers, particularly the subclass metal–organic frameworks (MOFs), are obtained by linking organic and inorganic units with solid bonds. More than 20,000 MOFs have been described and studied since they first appeared in the literature. They represent a promising new class of porous crystalline solids materials with amazing properties such as high surface capacities, large pore sizes, stable porosity, high thermal stability, and open channels with tunable sizes and topology. MOFs are rigid frameworks that can coordinate in 1D, 2D, or three dimensions and form highly porous networks. The organic units are usually di or polytopic carboxylates, interconnected with metallic centers, and produce supramolecular crystalline structures. The area of the MOFs surface range from 1000 to 10,000 m^2^/g [11,12,13]. Several synthesis routes have been used for MOFs synthesis. The slow evaporation method is a common method to prepare MOFs in which a solution of raw materials is concentrated by slow evaporation of the solvent at a fixed temperature, most often at room temperature.

The most used method is solvothermal synthesis, but other methods are used, including microwave-assisted synthesis, which allows the faster synthesis of crystals compared to conventional heating; electrochemical synthesis, when the metal ions are continuously inserted through an anodic dissolution to the reaction mixture that contains a conducting salt and linker molecules; the mechanochemical method, where mechanical breakage of intramolecular bonds takes place followed by a chemical transformation; the sonochemical technique, where well-known compounds are reproduced by these alternative routes [14,15,16,17,18,19,20].

MOFs are promising materials for applications in separation, adsorption catalysis [21,22,23,24], drug delivery agents, gas storage, corrosion, molecular recognition [25], antibacterial agents [26], sensors, and so on [27,28] because of their designable framework structures; the size, dimensions, shape, and surface functionality of the nanochannels can be tuned by changing the combination of metal ions and organic ligands.

In addition to di-, tri-, or tetradentate carboxylate ligands, phosphonates and sulfonates have been used as organic linkers to obtain MOFs. This field is relevant to the core concept of the metal–organic framework [29,30,31,32].

Diphosphinic acids have also been used for the synthesis of MOFs. They are similar to di-carboxylic acids. A porous tubular 1D-MOF, consisting of Cu(II), 1,2-bis(4-pyridyl)ethane, and P,P′-diphenyl-diphosphinate, was obtained in an easy and direct self-assembly process in either needle microcrystal or nanorod forms, depending on the reaction conditions [33].

1D structural arrays are obtained when P,P′-diphenylmethylenediphosphinic acid (H_2_pcp) reacts with alkaline metal ions such as Mg(SO_4_) or CaCl_2_·6H_2_O [34]. Using auxiliary ligands such as 2,2′-bipyridine, a new tetranuclear complex connected through water–hydrogen-bonding of zinc(II) with P,P′-diphenylmethylenediphosphinate was synthesized [35]. P,P′-diphenylmethylenediphosphinic (H_2_pcp), P,P′-diphenylethylenediphosphinic acids, and copper(II) salts were synthesized in the presence of 2,2′-bipyridine, and the effect of carbon chain length connecting the two phosphinate species, both in the free acids and in CuII/2,2′-bipyridine derivatives, were investigated [36].

The properties of heterocyclic phosphorus compounds and their complexations are being extensively studied for a wide variety of applications in numerous fields [37].

Phosphorus-bearing calixarenes have received significant attention for their ionophoric receptor characteristics and for their ability to localize transition metal reactive centers present close to the cavity as host–guest and supramolecular chemistry as “building blocks” three-dimensionally. The intention is to use them as selective cation receptors and delivery systems [38,39].

(2-Carboxyethyl)(phenyl)phosphinic acid represents another type of ligand used for obtaining metal–organic frameworks because it features polar phosphinic acid and carboxy moieties at the ends of a flexible –(CH_2_)n– spacer (n = 2), which is an efficient asymmetric ligand for inducing the formation of noncentrosymmetric structures for metal complexes [40,41]. 2-Carboxyethyl (phenyl)phosphinic acid has also been used as a flame retardant prepared via the reaction with melamine [42] or with dimethyl terephthalate and ethylene glycol [43] or with 2,5-furandicarboxylic acid and 1,6-hexanediol [44].

Only a few papers deal with the synthesis of metal networks in which 2-carboxyethyl(phenyl)phosphinic acid was involved, and this is the first one that describes this type of material as an adsorbent for heavy metals. Furthermore, the adsorption performance of CaCEPPA and CoCEPPA materials was investigated in the removal process of cadmium and lead ions from aqueous solutions. The optimum pH, concentration, and stirring time were investigated to reach the maximum adsorption capacities for both adsorbent materials used. Pseudo-first and second-order kinetic models were performed for fitting kinetic experimental data and Langmuir and Freundlich isotherms for modeling the equilibrium experimental data.

## 2. Results and Discussion

### 2.1. Characterization of Materials

**Figure 1:**
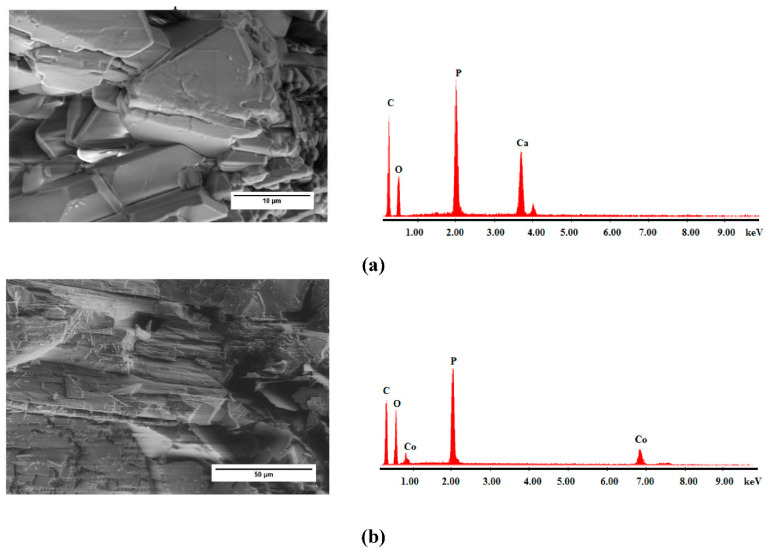
SEM images and EDX of the synthesized adsorbent materials (**a**) CaCEPPA; (**b**) CoCEPPA.

Figure 1 presents the morphology of the synthesized materials. Compared with CoCEPPA, whose surface is denser with particle conglomerates of several sizes and shapes, CaCEPPA based on Ca ions presents a well-ordered structure with particles of well-defined sizes and shapes.

**Figure 2:**
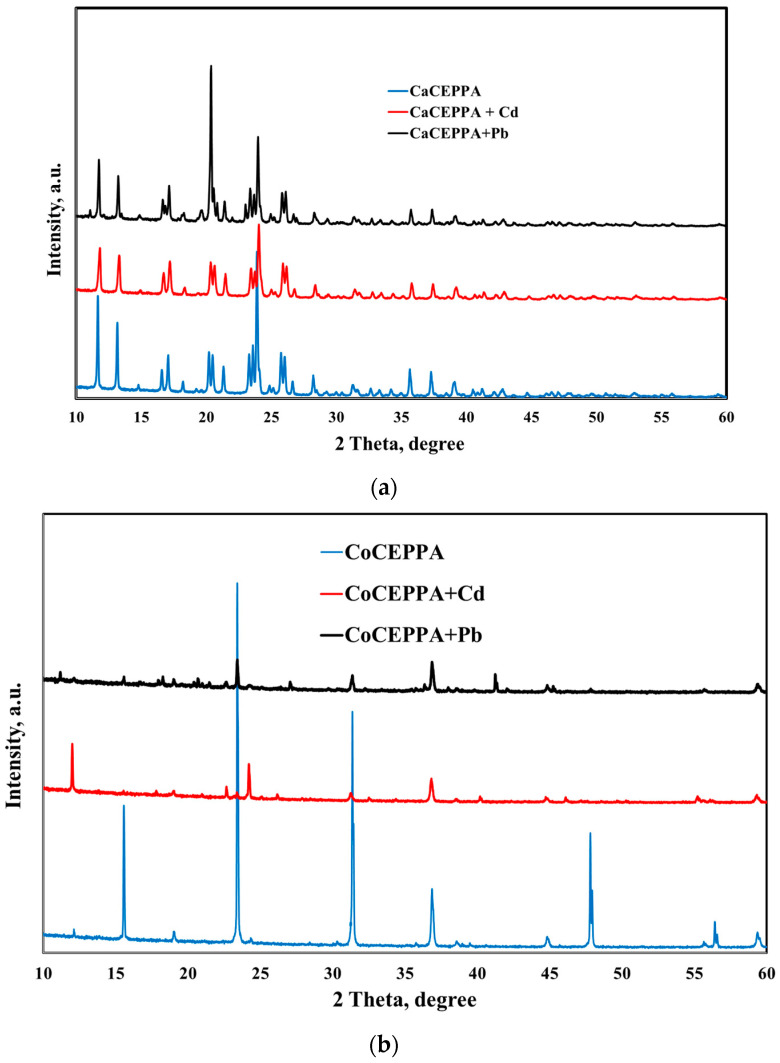
XRD patterns for (**a**) CaCEPPA; (**b**) CoCEPPA before and after adsorption of Cd^2+^ and Pb^2+^.

To investigate the crystalline structure of CoCEPPA and CaCEPPA before and after adsorption of lead and cadmium, PXRD measurements were performed, Figure 2.

**Figure 3:**
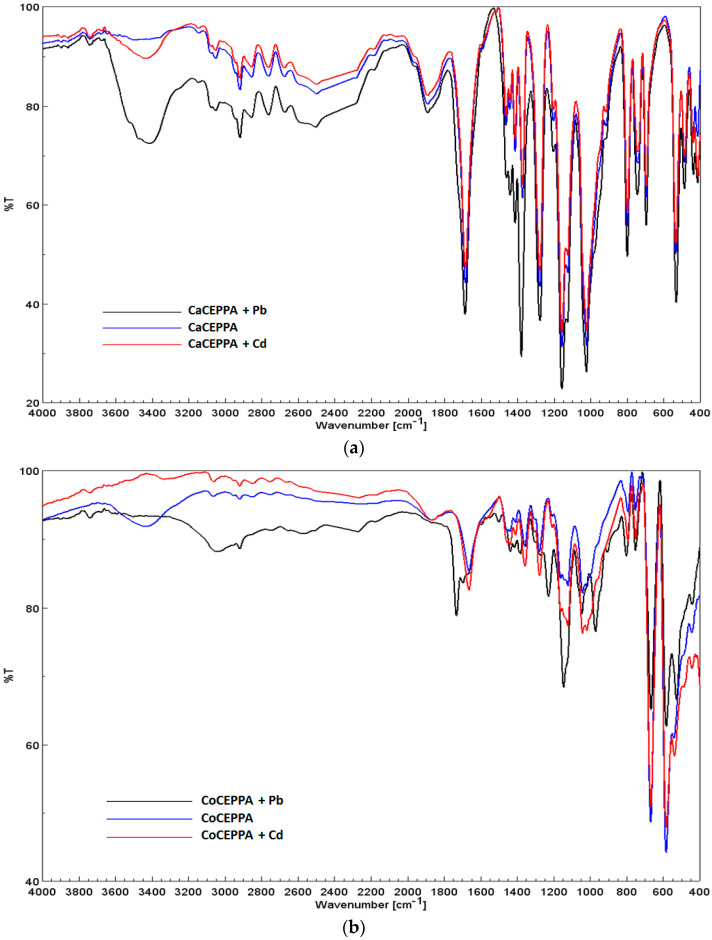
FT-IR spectra of CaCEPPA (**a**) and CoCEPPA (**b**).

Judging from the PXRD spectra of both adsorbent materials, the crystallinity is highly affected after the adsorbance of metal ions on the studied materials, in concordance with IR spectra of adsorbents before and after metal ions adsorption. The XRD analysis identified the formation of CdCO_3_ or Cd_3_(PO_4_)_2_, especially in the case of CoCEPPA adsorbent materials (Figure 2b), with two more peaks appearing at 22.67° and 24.19°. For CaCEPPA, these peaks are covered by the peaks specific to the based materials [45]. In the case of lead adsorption, it is also highlighted that some complexes are formed at the adsorbent surface due to the appearance of new peaks at 2θ = 11.1° and 16.6° [46] in the XRD spectrum of both studied materials. To gain a deeper understanding of the mechanism of adsorption, in addition to the isothermal and kinetic studies, FTIR spectra of Pb(II)-impregnated and Cd(II)-impregnated CoCEPPA and CaCEPPA were recorded and compared to that of pristine CoCEPPA and CaCEPPA (Figure 3a,b). The results indicate that chemisorption may be the main adsorption mechanism, with the carboxylic oxygen in both CoCEPPA and CaCEPPA potentially coordinating with the Pb^2+^ and Cd^2+^.

FTIR spectra of CaCEPPA compounds present a band at 1684 cm^−1^, which is absent in the spectra of CoCEPPA, revealing the presence of a carboxylic group in calcium-based adsorbent material. CoCEPPA bands in the region of 3500–3300 cm^−1^ (broad), together with the vibration band of H–O–H at 1638 cm^−1^, reveal the presence of H-bonded water in concordance with the crystallographic data [41]. While in the IR spectrum of CoCEPPA, no peak near 1700 cm^−1^ for the –COOH group is observed, two strong peaks around 1500–1345 cm^−1^ are observed, which are characteristic of absorption of asymmetric and symmetric vibrations of coordinated carboxylate groups present in the case of CaCEPPA. Strong characteristic bands of P=O in the region from 1390 to 1000 cm^−1^ and of P–O around 1050–850 cm^−1^ are present [46,47]. An increase in the peak strength or slight broadening of the existing peaks in the region 1700–1000 cm^−1^ are characteristic bands for P=O and of P–O, together with the appearance of new peaks created in the range of 400–600 cm^−1^ is related to Me^2+^-O peaks. All of these are evidence of coordination interactions between Me^2+^ and O atoms from the carboxylic group and indicate the involvement of the Cd^2+^ and Pb^2+^ on the surface of the adsorbent during the adsorption process. Results of FT-IR showed that the adsorption of Me^2+^ is accomplished by two processes, chemisorption and physisorption. The thermal stability studies of the CaCEPPA and CoCEPPA compounds were previously described [41].

### 2.2. Adsorption Studies

#### 2.2.1. Influence of Initial pH of Aqueous Solutions upon the Adsorption Properties of CaCEPPA and CoCEPPA Materials

The initial pH of the solution influences both phases involved in the adsorption processes, influencing the charge of the adsorbent surface and the form of the adsorbate.

**Figure 4:**
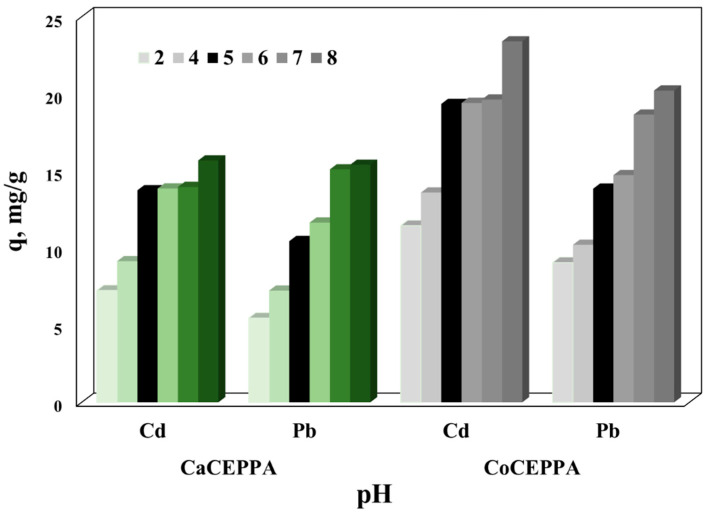
The influence of pH on the adsorption capacity of CaCEPPA and CoCEPPA in the removal process of cadmium and lead from aqueous solutions.

In addition to the physical adsorption in the pores of the adsorbent, it is always much better to favor the conditions for achieving strong electrostatic attractions. The experimental results regarding the influence of the initial pH of aqueous solutions containing 30 mg/L of cadmium and respective lead ions on the adsorption capacity of CaCEPPA and CoCEPPA are presented in Figure 4.

It can be observed that at lower pH values, due to the competition between the Cd^2+^/Pb^2+^ ions and the H^+^, the studied materials developed the lowest adsorption capacities [48]. Increasing the initial pH of aqueous solutions from 2 to 5 also increases the adsorption capacities of the studied materials. In the case of Cd ions removal, a significant increase at pH = 8 and in the case of Pb ions at pH > 6 could be observed, but in these cases, the removal could not be attributed to the adsorption process. In these cases, the removal is due to the precipitation of metal ions as hydroxides, according to the distribution diagram of cadmium and lead species function on pH [49,50]. To avoid the precipitation processes and to obtain the maximum adsorption capacities developed by the studied compounds, further adsorption studies were conducted using aqueous solutions containing metal ions with an initial pH of 5.

#### 2.2.2. Stirring Time Influence on Adsorption Properties of CaCEPPA and CoCEPPA Materials

**Figure 5:**
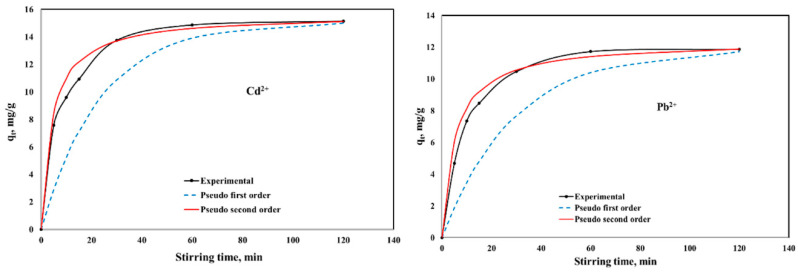
Kinetics of Cd^2+^ and Pb^2+^ adsorption onto CaCEPPA.

**Figure 6:**
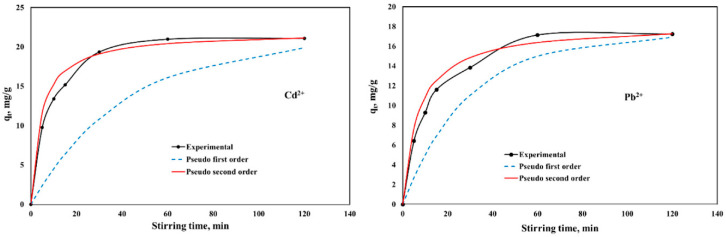
Kinetics of Cd^2+^ and Pb^2+^ adsorption onto CoCEPPA.

Figure 5 and Figure 6 present the influence of stirring time on the adsorption capacities of CaCEPPA and CoCEPPA in the removal process of Cd; respective Pb ions from aqueous solutions, and the non-linear pseudo-first-order and pseudo-second-order kinetic models were used for fitting the experimental data. The equilibrium between Cd, respective Pb ions, and CaCEPPA and CoCEPPA adsorbent materials was achieved in 60 min.

The pseudo-first-order and pseudo-second-order kinetic parameters, together with the error analysis results, are presented in Table 1.

From the experimental data presented in Table 1, it can be observed that for the pseudo-second-order kinetic model, values of the correlation coefficient close to unity and the lowest values of the chi-square test χ^2^ are obtained. The error functions ERRSQ and ARE obtained for the pseudo-second-order kinetic models present values of order of units; instead, for the pseudo-first-order kinetic model, these errors present values of order of hundreds. The results suggest that the adsorption of metal ions (Cd^2+^, respective Pb^2+^) onto CaCEPPA and CoCEPPA is best described by the pseudo-second-order kinetic model, implying a chemical sorption mechanism.

#### 2.2.3. Influence of Equilibrium Concentration of Metal Ions upon the Adsorption Properties of CaCEPPA and CoCEPPA Materials

**Figure 7:**
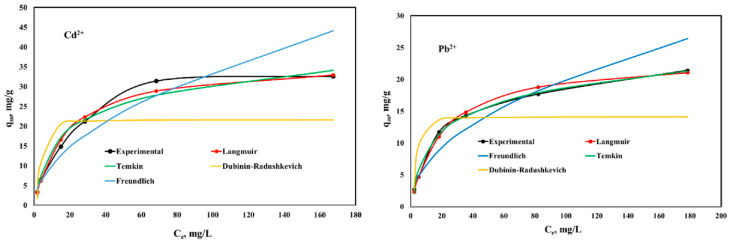
Equilibrium isotherms of Cd^2+^ and Pb^2+^ adsorption onto CaCEPPA.

**Figure 8:**
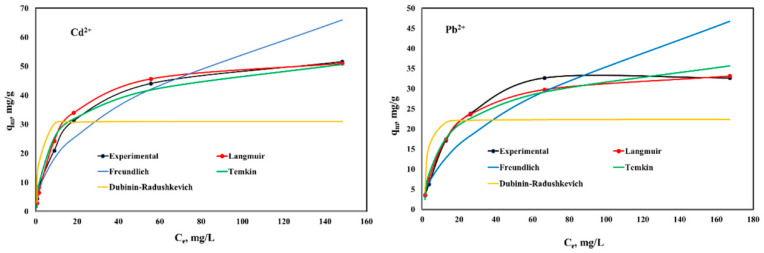
Equilibrium isotherms of Cd^2+^ and Pb^2+^ adsorption onto CoCEPPA.

The experimental results regarding the equilibrium studies and their fitting with the Langmuir, Freundlich, Temkin, and Dubinin–Raduschkevich isotherms are presented in Figure 7 and Figure 8.

It can be observed that both studied materials present a higher affinity for Cd^2+^ ions compared with Pb^2+^ ions. CoCEPPA developed a higher adsorption capacity for both metal ions compared with the adsorption capacities developed by CaCEPPA. The maximum adsorption capacity determined experimentally for CoCEPPA is 51.6 mg/g for Cd^2+^ and 32.7 mg/g for Pb^2+^, respectively. CaCEPPA developed a maximum adsorption capacity of 32.6 mg/g for Cd^2+^ ions removal and 21.4 mg/g for Pb^2+^, respectively.

The Langmuir, Freundlich, Temkin, and Dubinin–Raduschkevich isotherm parameters, together with the error analysis results, are presented in Table 2.

From the results presented in Table 2, the maximum adsorption capacities calculated from the Langmuir isotherm are very close to those determined experimentally for both studied materials, and both metal ions adsorbed could be observed. Additionally, in this case, the lowest values of the error functions and the highest values for the correlation coefficient are obtained, compared with the values obtained for the other studied isotherm. The Temkin isotherm model gave the best fit after the Langmuir isotherm, with an obtained correlation coefficient higher than 0.96, but the error values are higher than the Langmuir isotherm. The Temkin isotherm model, whose adsorption is characterized by a uniform distribution of the binding energies up to a notable maximum binding energy, was verified. The positive value of the variation of adsorption energy parameter b suggests that the adsorption of Cd^2+^/Pb^2+^ onto the studied materials is exothermic in nature (b = −ΔH). The values of theoretical monolayer saturation capacity in the Dubinin–Radushkevich model obtained using non-linear regression are all lower than the experimental amounts corresponding to the adsorption isotherm plateau, and the lower values obtained for the correlation coefficient and the highest value obtained for the studied errors indicate that the modeling of Dubinin–Radushkevich for the adsorption system of the Cd^2+^/Pb^2+^ is unacceptable. The obtained results clearly show that the values predicted from the Langmuir isotherm are close to the experimental data, suggesting that the Cd^2+^ and Pb^2+^ ions adsorption onto CaCEPPA and CoCEPPA materials take place under a homogeneous mechanism as a monolayer on the adsorbent surface. Therefore, by comparison, the order of the isotherm best fits the four sets of experimental data in this study is Langmuir > Temikn > Freundlich > Dubinin–Radushkevich.

**Figure 9:**
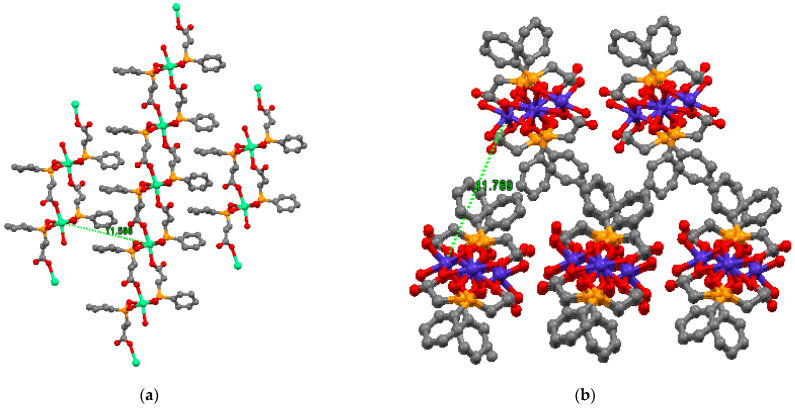
Packing of the CaCEPPA (**a**) and CoCEPPA (**b**).

To better understand the mechanism of the adsorption, we analyzed the packing of the CoCEPPA and CaCEPPA and measured the distances between the Me^2+^ situated in the adjacent layer. We found that the distance between metallic centers of two adjacent layers Co^2+^⋅⋅⋅Co^2+^ is 11.769Å, while for Ca^2+^⋅⋅⋅Ca^2+^, the distance is 11.566 Å, as can be observed in Figure 9. The 1D orientation of CoCEPPA compared with the layer structure of CaCEPPA allows the metal ions to more easily access the surface of the adsorbent material, and this explains the higher adsorption capacity of CoCEPPA [41].

Additionally, the properties of Pb^2+^ and Cd^2+^ are also important. The lowest Gibbs free energy of hydration was observed for Pb^2+^ (1425 kJ mol^−1^) in comparison with Cd^2+^ (1755 kJ mol^−1^). The radius of Cd^2+^ is 0.095 nm, while the radius of Pb^2+^ is 0.119 nm. Correlating the results of the adsorption studies with the XRD and FTIR analysis and the structure of the adsorbent materials, the complex mechanism involved in the metal ion removal is clear.

#### 2.2.4. Comparison with Other Adsorbents

The maximum adsorption capacity obtained from the Langmuir isotherm for the removal of Cd^2+^/Pb^2+^ ions onto the studied materials was compared with other findings in the literature on adsorption capacities and are presented in Table 3 and Table 4. It can be observed that the studied materials present a good adsorption efficiency in the removal of Cd^2+^/Pb^2+^ ions compared with similar materials presented in the specialty literature.

## 3. Materials and Methods

### 3.1. Materials

2-Carboxyethylphenylphosphinic acid (CEPPA) was purchased from Carbosynth Limited. Cobalt acetate tetrahydrate and calcium acetate monohydrate were purchased from Sigma-Aldrich. All chemicals were obtained from commercial sources and used without purification.

### 3.2. Instrumentation

Thermal analysis (TG-DTA) was performed on an SDT-Q600 analyzer from TA Instruments New Castle, DE, USA. A thermogravimetric analyzer (Perkin-Elmer, New York, NY, USA) was used at a 30–680 °C temperature range, at a heating rate of 10 °C/min, under an N_2_ flow. A FEG 250 microscope (Quanta, Field Electron and Iron Company (FEI), Hillsboro, OR, USA), equipped with an EDAX/ZAF quantifier, was used for obtaining SEM images. Lead and cadmium ions adsorption were measured using a SpectrAA 280 FS atomic absorption spectrophotometer (Varian, Melbourne, Australia). The adsorption studies were performed in batch mode using an SW23 shaker bath (Julabo Labortechnik GmbH, Seelbach, Germany).

### 3.3. Synthesis of Materials

#### Co_2_[(O_2_P(CH_2_CH_2_COO)(C_6_H_5_)(H_2_O)]_2_·2H_2_O (CoCEPPA) and Ca[O_2_P(CH_2_CH_2_COOH)(C_6_H_5_)]_2_ (CaCEPPA)

These compounds were synthesized similarly to our previous papers. A solution of Co(CH_3_COO)_2_·4H_2_O (50.0 mmol) or Ca(CH_3_COO)_2_·H_2_O (50.0 mmol) and bidistilled water (50 mL) was stirred at a constant speed of 1000 rpm until a clear (violet or white) solution was obtained. Secondly, 2-carboxyethylphenylphosphinic acid (CEPPA) (50.0 mmol) and bidistilled water (50 mL) were mixed at 60 °C until a colorless and clear solution was obtained. Both solutions were mixed, and the pH was adjusted to 2.8 with an aqueous solution of NaOH (0.1 M). Then, the clear white or violet solution was heated in an oil bath at 80 °C for 75 h, unperturbed. After 75 h heating, crystalline white (CaCEPPA) and black crystals (CoCEPPA) were isolated by filtration and, finally, air dried (yield: 62–70%) [55,56,57].

### 3.4. Adsorption Studies

The CaCEPPA and CoCEPPA materials were used as adsorbents in the removal of cadmium (Cd^2+^) and lead (Pb^2+^) ions from synthetic aqueous solutions to determine their adsorption properties. The process was conducted in batch mode, determining the influence of the initial pH (2–8) solution, stirring time (5–120 min), and initial concentrations of pollutants (C_0_ = 5–200 mg/L) upon the adsorption performance of the studied compounds. For all the studies, the ratio between the adsorbent and aqueous solution was 1:1 (a solution of 0.025 g adsorbent in 25 mL of water was used). The initial pH of solutions was calibrated at the desired values by the addition of 1M NaOH or HCl solutions and was measured with a Mettler Toledo pH meter. When the pH and the stirring time were varied, an aqueous solution containing 30 mg/L metal ions was used. When the pH and the initial concentration of ions were varied, the samples were stirred for 60 min. The initial and equilibrium concentration of cadmium and lead ions were determined using a Varian SpectrAA 280 FS atomic absorption spectrophotometer by atomic absorption spectroscopy. The adsorption performance of the studied materials was evaluated by calculating the adsorption capacity according to Equation (1):

(1) $$q_{e}=\frac{\left(C_{0}-C_{e}\right)\cdot V}{m}$$

where q_e_ is the adsorption capacity of the adsorbent at equilibrium (mg/g), V is the volume of the aqueous solution (L), C_0_ (mg/L) represents the initial concentration of metal ions (Cd or Pb) in the aqueous solutions, and C_e_ (mg/L) represents the residual concentration (the equilibrium concentration) of metal ions in solutions, and m is the mass of adsorbent (g).

The adsorption mechanism and the equilibrium between the adsorbents, cadmium, and lead ions, could be determined by matching the experimental results concerning the influence of stirring time on the adsorption capacity of CaCEPPA and CoCEPPA materials with the known kinetic models, pseudo-first-order and pseudo-second-order kinetic models, presented in their non-linear form in Equations (2) and (3) [57,58].

(2) $$q_{t}=q_{e}\left(1-\exp^{-k_{1}\cdot t}\right)$$

(3) $$q_{t}=\frac{k_{2}\cdot q_{e}^{2}\cdot t}{1+k_{2}\cdot q_{e}\cdot t}$$

where q_e_ and q_t_ (mg/g) is the adsorption capacity of the adsorbent at equilibrium and at a specific time t, k_1_ represents the rate constant of the pseudo-second-order kinetic model (min^−1^), k_2_ represents the rate constant of the pseudo-second-order kinetic model (g∙mg^−1^∙min^−1^), and t is the stirring time (min).

The maximum adsorption capacity of the studied materials was determined by modeling the experimental data, taking into account the influence of the initial concentration of metal ions from aqueous solutions on the adsorption behavior of CaCEPPA and CoCEPPA materials, using non-linear forms of Langmuir, Freundlich, Temkin, and Dubinin–Raduschkevich isotherms (Equations (4)–(7)) [57,58].

(4) $$q_{e}=\frac{q_{\max}\cdot K_{L}\cdot C_{e}}{1+K_{L}\cdot C_{e}}$$

where q_max_ (mg/g) is the maximum saturated monolayer adsorption capacity under the given conditions, K_L_ (L/mg) is the Langmuir constant related to the affinity between the adsorbent and adsorbate.

(5) $$q_{e}=K_{F}\cdot C_{e}^{\frac{1}{n}}$$

where K_F_ (mg/g)(L/mg)^1/n^ is the Freundlich constant and 1/n (0 < n < 10) is a Freundlich intensity parameter.

(6) $$q_{e}=\frac{\mathrm{RT}}{b}\ln\left(K_{T}C_{e}\right)$$

where R (J/mol K) is the gas constant, and T (K) is the absolute temperature, K_T_ is the adsorption potential or the equilibrium binding constant (Lg or Lmol^−1^) corresponding to the maximum binding energy and interaction between adsorbate and adsorbent, while adsorption energy, b_T_ (Jmol^−1^) is related to heat of adsorption (ΔQ), that is; b_T_ = ΔQ = −ΔH).

(7) $$q_{e}=q_{m}e^{-K_{\mathrm{DR}}\varepsilon^{2}}$$

where q_m_ (mg/g) is a constant in the Dubinin–Radushkevich isotherm model which is related to adsorption capacity; K_DR_ (mol^2^/kJ^2^) is a constant related to the mean free energy of adsorption.

(8) $$\varepsilon =\mathrm{RTln}\left(1+\frac{1}{C_{e}}\right)$$

where R (J/mol K) is the gas constant, and T (K) is the absolute temperature.

To predict the best kinetic and isotherm model which describes better the adsorption of cadmium and lead ions onto CaCEPPA and CoCEPPA materials, four error functions were applied: correlation coefficient R^2^; sum of squares of the errors ERRSQ (Equation (9)); chi-square test χ^2^ (Equation (10)); average relative error ARE (Equation (11)) [59,60,61,62].

(9) $$\mathrm{ERRSQ}=\overset{n}{\underset{i=1}{\sum }}{\left(q_{e\;\mathrm{calc}}-q_{e\;\exp}\right)}_{i}^{2}$$

(10) $$\chi^{2}=\overset{n}{\underset{i=1}{\sum }}\frac{{\left(q_{e\;\exp}-q_{e\;\mathrm{calc}\;}\right)}^{2}}{q_{e\;\mathrm{calc}}}$$

(11) $$\mathrm{ARE}=\frac{100}{n}\overset{n}{\underset{i=1}{\sum }}{\left|\frac{q_{e\;\mathrm{calc}}-q_{e\;\exp}}{q_{e\;\mathrm{calc}}}\right|}_{i}$$

## 4. Conclusions

CaCEPPA and CoCEPPA showed acceptable performance in the removal of Cd^2+^ and Pb^2+^ from an aqueous solution. The metal ions’ adsorption onto the studied materials is better described by the pseudo-second-order kinetic model and by the Langmuir isotherm, suggesting that the adsorption mechanism could be chemical sorption in the case of Me^2+^ ions. CoCEPPA developed the highest adsorption capacity compared to CaCEPPA, and both materials presented a higher affinity for Cd ions compared to Pb^2+^ ions. In addition to physical adsorption, the phenyl functional groups of the adsorbent materials, which attract the metal ions through electrostatic interactions or Me-O bonds, have a great contribution. This explains the better adsorption performance developed by the CoCEPPA, which is a zig-zag chain 1D structure with available functional groups for adsorption on the entire length of the chain. In the case of CaCEPPA, a monoclinic system, due to the fact that phenyl groups are orientated into the interlayer space, the metal ions do not easily reach them to form a Me-O bond; therefore, less adsorption performance is developed by this material.

## Figures and Tables

**Table 1:** Pseudo-first-order and pseudo-second-order kinetic parameters for Cd^2+^ and Pb^2+^ adsorption from aqueous solutions onto CaCEPPA and CoCEPPA materials.

Kinetic Model	Parameters	CaCEPPA		CoCEPPA	
		Cd	Pb	Cd	Pb
	q_e exp_, mg/g	15.14	11.85	21.08	17.23
Pseudo-first-order	q_e calc_, mg/g	7.15	5.545	8.56	9.44
	k_1_, min^−1^	0.0424	0.0344	0.024	0.0342
	R^2^	0.9674	0.8932	0.7840	0.8573
	ERRSQ	353	221	970	331
	χ^2^	99.5	84.7	318	74.7
	ARE, %	228	253	458	183
Pseudo-second-order	q_e calc_, mg/g	15.67	12.4	21.92	18.25
	k_2_, g mg^−1^ min^−1^	0.0149	0.0153	0.0104	0.0079
	R^2^	0.9975	0.9959	0.9968	0.9928
	ERRSQ	4.21	3.09	10.4	6.39
	χ^2^	0.389	0.45	0.727	0.59
	ARE, %	5.81	7.31	7.14	8.26

**Table 2:** Langmuir, Freundlich, Temkin, and Dubini–Raduschkeich isotherm parameters for Cd^2+^ and Pb^2+^ adsorption from aqueous solutions onto CaCEPPA and CoCEPPA materials.

Isotherm Model	Parameters	CaCEPPA		CoCEPPA	
		Cd	Pb	Cd	Pb
	q_m exp_, mg/g	32.6	21.4	51.6	32.7
Langmuir	q_m calc_, mg/g	36.5	23.52	54.9	35.8
	k_L_, L/mg	0.0556	0.0483	0.0886	0.0739
	R^2^	0.9964	0.9968	0.9909	0.9968
	ERRSQ	10.6	2.29	27.3	10.6
	χ^2^	0.46	0.21	2.1	0.54
	ARE, %	5.3	5.99	18.1	5.78
Freundlich	k_F_, (mg/g) (L/mg)^n^	3.106	2.188	6.65	3.509
	1/n	0.5183	0.4805	0.4590	0.5067
	R^2^	0.9475	0.9448	0.9648	0.9193
	ERRSQ	165	38.5	253	259
	χ^2^	4.86	2.38	5.23	8.01
	ARE, %	17.3	15.3	14.6	22.3
Temkin	b, J/mol	353	563	275	352
	K_T_, L/g	0.768	0.713	1.88	0.44
	R^2^	0.9700	0.9931	0.9801	0.9628
	ERRSQ	22.9	1.83	36.0	29.9
	χ^2^	1.82	0.422	7.59	1.85
	ARE, %	18.5	9.36	42.6	16.3
Dubinin–Raduschkevich	q_m_, mg/g	21.6	14.1	31.0	22.3
	K_DR_, mol^2^/J^2^	2 × 10^−6^	2 × 10^−6^	4 × 10^−7^	1 × 10^−6^
	R^2^	0.7985	0.7752	0.7785	0.7630
	ERRSQ	269	95.3	746	324
	χ^2^	14.8	7.58	25.9	16.5
	ARE, %	43.8	24.6	37.7	34.0

**Table 3:** Comparison of the maximum adsorption capacities given/demonstrated by similar adsorbents in the treatment process of aqueous solutions containing Cd^2+^ ions.

Adsorbent	q_m_ (mg g^−1^)	References
Functionalized Zr-MOFs	41.32	[10]
UiO-66-NHC(S)NHMe	49	[51]
TMU-5	43	[52]
Cu-DPA MOF	1.334	[53]
LDH/MOF NC	415.3	[54]
CaCEPPA	32.6	This paper
CoCEPPA	51.6	

**Table 4:** Comparison of the maximum adsorption capacities given/demonstrated by similar adsorbents in the treatment process of aqueous solutions containing Pb^2+^ ions.

Adsorbent	q_m_ (mg g^−1^)	References
Functionalized Zr-MOFs	50.51	[10]
UiO-66-NHC(S)NHMe	232	[51]
TMU-5	251	[52]
Cu-DPA MOF	2.19	[53]
LDH/MOF NC	301.4	[54]
CaCEPPA	21.4	This paper
CoCEPPA	32.7	

## Data Availability

All data analyzed during this study are included in this published article.

## References

[1] Dhahri R., Yılmaz M., Mechi L., Alsukaibi A.K.D., Alimi F., ben Salem R., Moussaoui Y. (2022). Optimization of the Preparation of Activated Carbon from Prickly Pear Seed Cake for the Removal of Lead and Cadmium Ions from Aqueous Solution. Sustainability.

[2] Flora G., Gupta D., Tiwari A. (2012). Toxicity of lead: A review with recent updates. Interdiscip. Toxicol..

[3] Maranescu B., Lupa L., Visa A. (2016). Synthesis, characterizations and Pb(II) sorption properties of cobalt phosphonate materials. Pure Appl. Chem..

[4] Skipper A., Sims J.N., Yedjou C.G., Tchounwou P.B. (2016). Cadmium chloride induces DNA damage and apoptosis of human liver carcinoma cells via oxidative stress. Int. J. Environ. Res. Public Health.

[5] Xu M.Y., Wang P., Sun Y.J., Yi-Jun Wu Y.J. (2019). Disruption of kidney metabolism in rats after subchronic combined exposure to low-dose cadmium and chlorpyrifos. Chem. Res. Toxicol..

[6] Abbas A., Al-Amer A.M., Laoui T., Al-Marri M.J., Nasser M.S., Khraisheh M., Atieh M.A. (2016). Heavy metal removal from aqueous solution by advanced carbon nanotubes: Critical review of adsorption applications. Sep. Purif. Technol..

[7] Marques J.P., Vaz C.M.P., Sígolo J.B., Rodrigues V.G.S. (2022). Soils of the Ribeira Valley (Brazil) as Environmental Protection Barriers: Characterization and Adsorption of Lead and Cadmium. Sustainability.

[8] Kim H., Lee B.I., Byeon S.H. (2015). The inner filter effect of Cr (VI) on Tb–doped layered rare earth hydroxychlorides: New fluorescent adsorbents for the simple detection of Cr (VI). Chem. Commun..

[9] Lin R.B., Li T.Y., Zhou H.L., He C.T., Zhang J.P., Chen X.M. (2015). Tuning fluorocarbon adsorption in new isoreticular porous coordination frameworks for heat transformation applications. Chem. Sci..

[10] Wang K., Gu J., Yin N. (2017). Efficient removal of Pb(II) and Cd(II) Using NH_2_-Functionalized Zr-MOFs via rapid microwave-promoted synthesis. Ind. Eng. Chem. Res..

[11] Wang Z., Cohen S.M. (2009). Postsynthetic modification of metal-organic frameworks. Chem. Soc. Rev..

[12] Furukawa H., Cordova K.E., O’Keeffe M., Yaghi O.M. (2013). The Chemistry and Applications of Metal-Organic Frameworks. Science.

[13] Hu M. (2011). Design, Synthesis and Applications of Metal Organic Frameworks. Ph.D. Thesis.

[14] Sun Y., Zhou H.C. (2015). Recent progress in the synthesis of metal–organic frameworks. Sci. Technol. Adv. Mater..

[15] Stock N., Biswas S. (2012). Synthesis of Metal-Organic Frameworks (MOFs): Routes to Various MOF Topologies, Morphologies, and Composites. Chem. Rev..

[16] Macarie L., Simulescu V., Ilia G. (2019). Ultrasonic irradiation used in synthesis of aminophosphonates. Monatsh. Chem..

[17] Khan M.A., Jhung S.H. (2015). Synthesis of metal-organic frameworks (MOFs) with microwave or ultrasound: Rapid reaction, phase-selectivity, and size reduction. Coord. Chem. Rev..

[18] Iliescu S., Ilia G., Pascariu A., Popa A., Plesu N. (2006). Novel synthesis of phosphorus containing polymers under inverse phase transfer catalysis. Polymer.

[19] Visa A., Mracec M., Maranescu B., Maranescu V., Ilia G., Popa A., Mracec M. (2012). Structure simulation into a lamellar supramolecular network and calculation of the metal ions/ligands ratio. Chem. Cent. J..

[20] Popa A., Ilia G., Pascariu A., Iliescu S., Plesu N. (2005). Grafted styrene-divinylbenzene copolymers containing benzaldehyde and their Wittig reactions with various phosphonium salts. Chin. J. Polym. Sci..

[21] Maranescu B., Lupa L., Visa A. (2018). Heavy metal removal from waste waters by phosphonate metal organic frameworks. Pure Appl. Chem..

[22] Nistor M.A., Muntean S.G., Maranescu B., Visa A. (2020). Phosphonate metal organic frameworks used as dyes removal materials from wastewaters. Appl. Organomet. Chem..

[23] Maranescu B., Popa A., Lupa L., Maranescu V., Visa A. (2018). Use of chitosan complex with aminophosphonic groups and cobalt for the removal of Sr^2+^ ions. Sep. Sci. Technol..

[24] Lupa L., Maranescu B., Visa A. (2018). Equilibrium and kinetic studies of chromium ions adsorption on Co(II) based phosphonate metal organic frameworks. Sep. Sci. Technol..

[25] Maranescu B., Lupa L., Tara Lunga Mihali M., Plesu N., Maranescu V., Visa A. (2018). The Corrosion Inhibitor Behavior of Iron in Saline Solution by the Action of Magnesium Carboxyphosphonate. Pure Appl. Chem..

[26] Popa A., Ilia G., Iliescu S., Dehelean G., Pascariu A., Bora A., Pacureanu L. (2004). Mixed quaternary ammonium and phosphonium salts bound to macromolecular supports for removal bacteria from water. Mol. Cryst. Liq. Cryst..

[27] Uemura T., Yanai N., Kitagawa S. (2009). Polymerization reactions in porous coordination polymers. Chem. Soc. Rev..

[28] Sharmin E., Zafar F. (2016). Introductory Chapter: Metal Organic Frameworks (MOFs). Metal-Organic Frameworks.

[29] Shimizu G.K.H., Vaidhyanathan R., Taylor J.M. (2009). Phosphonate and sulfonate metal organic frameworks. Chem. Soc. Rev..

[30] Clearfield A., Demadis K.D. (2012). Metal Phosphonate Chemistry: From Synthesis to Applications.

[31] Maranescu B., Visa A., Mracec M., Ilia G., Maranescu V., Simon Z., Mracec M. (2011). Lamellar Co2+ vinylphosphonate metal organic framework.PM3 semi-empirical analysis of structural properties. Rev. Roum. Chim..

[32] Maranescu B., Visa A., Ilia G., Simon Z., Demadis K., Colodrero R.M.P.A., Vallcorba O., Rius J., Choquesillo-Lazarte D. (2014). Synthesis and characterization of styryl phosphonic acid and its use as new ligand for phosphonate metal organic framework. J. Coord. Chem..

[33] Bataille T., Bracco S., Comotti A., Costantino F., Guerri A., Ienco A., Marmottini F. (2012). Solvent dependent synthesis of micro- and nano- crystalline phosphinate based 1D tubular MOF: Structure and CO_2_ adsorption selectivity. Cryst. Eng. Comm..

[34] Midollini S., Lorenzo-Luis P., Orlandini A. (2006). Inorganic–organic hybrid materials of p,p0-diphenylmethylenediphosphinic acid (H_2_pcp) with magnesium and calcium ions: Synthesis and characterization of [Mg(Hpcp)_2_], [Mg(Hpcp)_2_(H_2_O)_4_], [Mg(pcp)(H_2_O)_3_](H_2_O), [Ca(Hpcp)_2_] and [Ca(pcp)(_H2O_)] complexes. Inorg. Chim. Acta.

[35] Ienco A., Midollini S., Orlandini A., Costantino F. (2007). Synthesis and Structural Characterization of a Tetranuclear Zinc(II) Complex with *P,P*′ diphenylmethylenediphosphinate (pcp) and2,2′-Bipyridine (2,2′-bipy) Ligands. Z. Naturforsch..

[36] Costantino F., Ienco A., Midollini S., Orlandini A., Sorace L., Vacca A. (2008). Copper(II) complexes with bridging diphosphinates—The effect of the elongation of the aliphatic chain on the structural arrangements around the metal centres. Eur. J. Inorg. Chem..

[37] Plinta H.J., Neda I., Schmutzler R. (1994). 1.3-Dimethyl-l,3-diaza-2-R-5,6-benzo-2 λ3-phosphorinan-4-ones (R = F, Me2N, 2-Methylpiperidino, MeC(:O)NH-) as Ligands in Transition-Metal Complexes; Synthesis and Structure of DichloroPlatinum(II)- and Tetracarbonyl-Metal(0) Coordination Compounds (Metal = Cr, Mo and W). Z. Naturforsch..

[38] Dieleman C.B., Matt D., Neda I., Schmutzler R., Harriman A., Yaftian R. (1999). Hexahomotrioxacalix[3]arene: A scaffold for a C3-symmetric phosphine ligand that traps a hydridorhodium fragment inside a molecular funnel. Chem. Commun..

[39] Vollbrecht A., Neda I., Thonnessen H., Jones P.G., Harris R.K., Crowe L.A., Schmutzler R. (1997). Chemische Synthesis, structure, and reactivity of tetrakis(o,o-phosphorus)-bridged calix[4]resorcinols and their derivatives. Berichte.

[40] Hu Q.S., Zhang X.Z., Luo S.F., Sun Y.H., Du Z.-Y. (2011). Two polymorphs of (2-carboxyethyl)-(phenyl)phosphinic acid. Acta Crystallogr..

[41] Bazaga-García M., Vílchez-Cózar Á., Maranescu B., Olivera-Pastor P., Marganovici M., Ilia G., Cabeza Díaz A., Visa A., Colodrero R.M.P. (2021). Synthesis and electrochemical properties of metal(II)-carboxyethylphenylphosphinates. Dalton Trans..

[42] You L., Hui Y., Shi X., Peng Z. (2012). Study on the synthesis and characterization of a novel phosphorus-nitrogen containing intumescent flame retardant. Adv. Mater. Res..

[43] Li L.J., Duan R.T., Zhang J.B., Wang X.L., Chen L., Yu-Zhong W. (2013). Phosphorus-Containing Poly(ethylene terephthalate): Solid-State Polymerization and Its Sequential Distribution. Ind. Eng. Chem. Res..

[44] Wang G., Jiang M., Zhang Q., Wang P., Qu X., Zhou G. (2018). Poly(hexamethylene 2,5-furandicarboxylate) copolyesters containing phosphorus: Synthesis, crystallization behavior, thermal, mechanical and flame retardant properties. Polym. Degrad. Stab..

[45] YiYi D., Huang S., Laird D.A., Wang X., Dong C. (2018). Quantitative mechanisms of cadmium adsorption on rice straw- and swine manure-derived biochars. Environ. Sci. Pollut. Res..

[46] Sharma R., Sarswat A., Pittman C.U., Mohan D. (2017). Cadmium and lead remediation using magnetic and non-magnetic sustainable biosorbents derived from Bauhinia purpurea pods. RSC Adv..

[47] Xue C.C., Li M.X., Shao M. (2016). Two novel 2D cadmium compounds with noncentrosymmetric or symmetric network dependent on different pH values. Russ. J. Coord. Chem..

[48] Wang X., Wang L., Wang Y., Tan R., Ke X., Zhou X., Cheng J., Hou H., Zhou M. (2017). Calcium sulfate hemihydrate whiskers obtained from flue gas desulfurization gypsum and used for the adsorption removal of lead. Crystals.

[49] Liang Y., Jun M., Liu W. (2007). Enhanced removal of lead(II) and cadmium(II) from water in alum coagulation by ferrate(VI) pretreatment. Water Environ. Res..

[50] Yang T., Sheng L., Wang Y., Wyckoff K.N., He C., He Q. (2018). Characteristics of Cadmium Sorption by Heat-Activated Red Mud in Aqueous Solution. Sci. Rep..

[51] Abbasi A., Moradpour T., Van Hecke K. (2015). A new 3D cobalt (II) metal–organic framework nanostructure for heavy metal adsorption. Inorg. Chim. Acta.

[52] Rahimi E., Mohaghegh N. (2016). Removal of Toxic Metal Ions from Sungun Acid Rock Drainage Using Mordenite Zeolite, Graphene Nanosheets, and a Novel Metal–Organic Framework. Mine Water Environ..

[53] Waritu H.H., Aregahegn D.A., Abdisa C.M., Minaleshewa A. (2022). High Performance Copper Based Metal Organic Framework for Removal of Heavy Metals From Wastewater. Front. Mater..

[54] Soltani R., Pelalak R., Pishnamazi M., Marjani A., Albadarin A.B., Sarkar M.S., Shirazian S. (2021). A novel and facile green synthesis method to prepare LDH/MOF nanocomposite for removal of Cd(II) and Pb(II). Sci Rep..

[55] Visa A., Plesu N., Maranescu B., Ilia G., Borota A., Crisan L. (2021). Combined Experimental and Theoretical Insights into the Corrosion Inhibition Activity on Carbon Steel Iron of Phosphonic Acids. Molecules.

[56] Visa A., Maranescu B., Bucur A., Iliescu S., Demadis K. (2014). Synthesis and characterization of a novel phosphonate metal organic framework starting from copper salts. Phosphorus Sulfur Silicon Relat. Elem..

[57] Visa A., Maranescu B., Lupa L., Crisan L., Borota A. (2020). New Efficient Adsorbent Materials for the Removal of Cd(II) from Aqueous Solutions. Nanomaterials.

[58] Lin J., Wang L. (2009). Comparison between linear and non-linear forms of pseudo-first-order and pseudo-second-order adsorption kinetic models for the removal of methylene blue by activated carbon. Front. Environ. Sci. Eng. China.

[59] Kumar K.V., Porkodi K., Rocha F. (2008). Comparison of various error functions in predicting the optimum isotherm by linear and non-linear regression analysis for the sorption of basic red 9 by activated carbon. J. Hazard. Mater..

[60] Obayomi K.S., Bello J.O., Yahya M.D., Chukwunedum E., Adeoye J.B. (2020). Statistical analyses on effective removal of cadmium and hexavalent chromium ions by multiwall carbon nanotubes (MWCNTs). Heliyon.

[61] Tǎmaş A., Cozma I., Cocheci L., Lupa L., Rusu G. (2020). Adsorption of orange II onto Zn_2_Al–layered double hydroxide prepared from zinc. Ash. Front. Chem..

[62] Azmi S.N.H., Al Lawati W.M., Al Hoqani U.H.A., Al Aufi E., Al Hatmi K., Al Zadjali J.S., Rahman N., Nasir M., Rahman H., Khan S.A. (2022). Development of a Citric-Acid-Modified Cellulose Adsorbent Derived from *Moringa peregrina* Leaf for Adsorptive Removal of Citalopram HBr in Aqueous Solutions. Pharmaceuticals.

